# Ultrasound Training in the Emergency Medicine Clerkship

**DOI:** 10.5811/westjem.2015.9.27290

**Published:** 2015-11-12

**Authors:** Mark Favot, Cheryl Courage, Jacob Mantouffel, David Amponsah

**Affiliations:** * Wayne State University School of Medicine, Department of Emergency Medicine, Detroit, Michigan; † Henry Ford Health System, Department of Emergency Medicine, Detroit, Michigan

## Abstract

**Introduction:**

The curriculum in most emergency medicine (EM) clerkships includes very little formalized training in point-of-care ultrasound. Medical schools have begun to implement ultrasound training in the pre-clinical curriculum, and the EM clerkship is an appropriate place to build upon this training. The objectives are (1) to evaluate the effectiveness of implementing a focused ultrasound curriculum within an established EM clerkship and (2) to obtain feedback from medical students regarding the program.

**Methods:**

We conducted a prospective cohort study of medical students during an EM clerkship year from July 1, 2011, to June 30, 2012. Participants included fourth-year medical students (n=45) enrolled in the EM clerkship at our institution. The students underwent a structured program focused on the focused assessment with sonography for trauma exam and ultrasound-guided vascular access. At the conclusion of the rotation, they took a 10-item multiple choice test assessing knowledge and image interpretation skills. A cohort of EM residents (n=20) also took the multiple choice test but did not participate in the training with the students. We used an independent samples *t*-test to examine differences in test scores between the groups.

**Results:**

The medical students in the ultrasound training program scored significantly higher on the multiple-choice test than the EM residents, *t*(63)=2.3, *p*<0.05. The feedback from the students indicated that 82.8% were using ultrasound on their current rotations and the majority (55.2%) felt that the one-on-one scanning shift was the most valuable aspect of the curriculum.

**Discussion:**

Our study demonstrates support for an ultrasound training program for medical students in the EM clerkship. After completing the training, students were able to perform similarly to EM residents on a knowledge-based exam.

## INTRODUCTION

Often, the only opportunity medical students have to spend a significant amount of time in the emergency department (ED), caring for acutely ill, undifferentiated patients is during the emergency medicine (EM) clerkship,^1^ which typically take place exclusively during the fourth-year of medical school. In addition to EM, medical students in EM clerkships will do their residency training in a variety of specialities such as surgery, internal medicine, and obstetrics/gynecology, each of which uses point-of-care ultrasound (POCUS) in their practice.^2^–^5^ However, given the current structure of the clinical clerkship curriculum at the majority of medical schools, students are not routinely exposed to POCUS.^6^ In fact, the majority of medical students receive no formal education in ultrasound during medical school,^7^,^8^ and in the instances when they do receive training, it is unclear if they develop competency.^9^

The Accreditation Council for Graduate Medical Education (ACGME) guidelines for specialty training list POCUS applications as a requirement of nearly every residency program.^2^–^5^,^10^ Since many residents struggle to gain competence in POCUS, we believe that the earlier and more often training is implemented into the undergraduate medical education curricula, the more likely these students will be proficient upon completion of their residency training. A study of first-year medical students demonstrated that after training they could assess the abdominal aorta of healthy volunteers to the same standard achieved by professional sonographers.^11^ These students received just four hours of formal instruction from a single physician in a small group setting, giving encouragement to educators that effective ultrasound education to students does not require a burdensome commitment of resources.

There are many factors outside of the students’ control that interfere with the opportunity to gain experience with POCUS in the ED, including the fast pace of care, high volume and higher acuity of patients. Given these barriers, it is common for students to complete an EM rotation with limited experience in this rapidly growing field.^12^,^13^ It is therefore imperative that medical students gain experience with this modality during formalized periods of instruction, in addition to exposure during their ED shifts. The EM clerkship provides an excellent opportunity to fill this gap in the undergraduate medical curriculum. The purposes of this study were (1) to evaluate the effectiveness of implementing a focused ultrasound curriculum within an established EM clerkship and (2) to obtain feedback from medical students regarding the program.

## METHODS

We conducted a prospective static-group comparison study of medical students during an EM clerkship conducted from July 1, 2011 to June 30, 2012.

### Setting

The setting was a large, urban, academic medical center, a Level 1 trauma center with an annual ED census of approximately 96,000 patients. The hospital has a large EM residency program (n=42 categorical residents), as well as combined residency programs in EM/Internal Medicine and EM/Internal Medicine/Critical Care Medicine. The department also has a fellowship in emergency ultrasound. We obtained institutional review board approval, and a waiver of informed consent was granted as the project was a part of an educational curriculum and participation was voluntary.

### Participants

**Figure 1:**
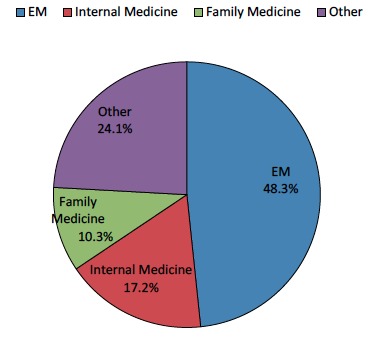
Medical students’ identified specialty of interest. *EM,* emergency medicine

Participants included fourth-year medical students (n=45) in the EM clerkship during the 2011–2012 academic year. Participation was optional, and lack of participation had no impact on clerkship grade. Students were excluded from participating if they were unable to attend the introductory training session. Figure 1 contains information regarding the chosen specialties of the students. Approximately half of the students identified themselves as pursuing a career in EM, while internal medicine and family medicine were the next most popular choices.

Due primarily to time constraints (one year for data collection) and the small number of rotating medical students, a control group consisting of medical students was not feasible; therefore, we used a non-randomized sample of EM residents (n=20) to serve as a comparison group. Members of the comparison group did not partake in any of the curricular components of the program, but each of them had received formal training in ultrasound during their residency, including an introductory course in emergency ultrasound during intern year. The concepts taught to the students during the didactic and hands-on components of the curriculum were also taught to the residents during their intern year as part of the residency curriculum.

### Methods of Instruction

A POCUS curriculum for EM clerkship students was developed by the faculty of the Emergency ultrasound fellowship program. The curriculum centered on the focused assessment with sonography for trauma (FAST) exam and ultrasound-guided vascular access. We felt that these applications were most relevant to the student due to their frequent use in clinical practice. Methods of instruction included didactic presentations, hands-on ultrasound scanning of live human models and tissue phantoms, and a dedicated ultrasound scanning shift working one-on-one with the ultrasound fellow in the ED.

After orientation on the first day of the clerkship, participants attended a two-hour instructional session on the FAST exam and ultrasound-guided vascular access techniques. This introductory session was led by the fellow and incorporated didactic lecture and hands-on practice in our institution’s simulation center. The didactic session lasted approximately 75 minutes, with 50 minutes devoted to the FAST exam and 25 minutes to vascular access. The ultrasound machines used in the simulation center were a GE Logiq E and a GE Venue 40 (Milwaukee, WI). Each student was required to perform a minimum of two ultrasound-guided vascular access procedures, one on a peripheral vessel phantom, and one on a central venous access mannequin, as well as two FAST exams.

Following this session, students were assigned a dedicated ultrasound shift in the ED to reinforce the techniques learned in the simulation center. During these shifts all students were allowed the opportunity to perform multiple FAST examinations and attempt ultrasound-guided, peripheral venous access procedures on ED patients. Throughout the clerkship, the students were able to perform FAST exams on all trauma activations. Additionally, students had the opportunity to place both peripheral and central lines in the course of their clerkship, many of which were done with ultrasound guidance, thus allowing further opportunity for skill refinement.

### Impact/Effectiveness

#### Assessment

A 10-item multiple choice test was developed to test students’ knowledge on the FAST exam and ultrasound-guided vascular access. Items and content on the test had been used previously during the pre-clinical POCUS training that the emergency ultrasound fellowship faculty has led for the past six years.^14^,^15^ The test was administered to the students individually during the last week of the clerkship under the supervision of the principle investigator. The EM residents were given the same test during the mid-point of the study period. Residents were approached by the investigators during a regularly scheduled conference and asked to participate. Participation by residents was voluntary and did not impact status within the program. The residents completed the test simultaneously under the supervision of the principle investigator. To ensure responses were anonymous, we collected only residents’ level of training and test responses.

One month after the conclusion of the clerkship, each student was contacted by the primary investigator by email and asked to respond to a brief, seven-item survey regarding their ultrasound training experience. Survey questions are presented in Table 1.

#### Statistical Analysis

We used an independent samples t-test to examine differences in test scores between students and residents. We performed all analyses with IBM SPSS 20.

## RESULTS

**Figure 2:**
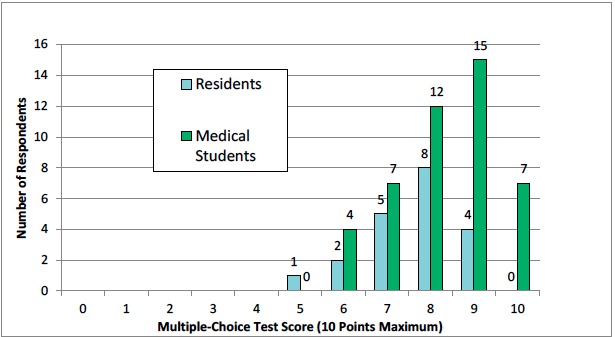
Test scores of medical students and residents.

The mean test score for the medical student participants in the ultrasound curriculum was 8.3 (SD 1.2) and the mean test score for the residents in the comparison group was 7.6 (SD 1.1). This difference in score between the two groups was statistically significant (p=0.026). Table 2 and Figure 2 show additional details of the test scores for the medical students and the residents.

The results from the feedback survey administered to the students are presented in Table 1. The survey response rate was 64% (n=29). Most of the students stated that they were using ultrasound on current or subsequent rotations (82.8%) and planned to use it during their residency (93.1%). A majority of the students replied that they valued the one-on-one instruction (55.2%) and would choose to add more than one scanning shift (51.7%) when asked how the curriculum could be improved. Of different ultrasound applications, students most wanted additional experience with bedside echocardiography (24.1%).

## DISCUSSION

The current study demonstrates that medical students are able to perform similarly to EM residents on a multiple-choice examination following the completion of an EM clerkship with a focused POC ultrasound curriculum.

Few studies of medical students performing ultrasound have demonstrated competence either in terms of knowledge or in skills such as image acquisition or interpretation. In a study with first-year medical students incorporating an objective, knowledge-based ultrasound test and a practical hands-on examination, investigators compared students’ performance pre- and post-educational intervention, randomizing students into two groups, “early” and “late” intervention.^10^ Both groups demonstrated improved performance after the intervention, yet they did not compare performance with a group of subjects who had already achieved some level of competence in POCUS. The pre- and post-test method of determining whether medical students gained knowledge is used frequently in the literature in training undergraduate medical students in POCUS.^16^,^17^

Another education research method frequently encountered in the literature is the assessment of the confidence and satisfaction of the learners after they have completed the curriculum.^16^,^18^,^19^ We feel that the follow-up survey responses we obtained offer particularly high-yield insights to educators looking to introduce POCUS curricula into existing EM clerkships. The feedback not only shows high levels of satisfaction but also offers potential course improvements, such as including more than one scanning shift and adding other applications such as POC echocardiography to the program. Thus, clerkship directors at programs with core ultrasound faculty could replicate our curriculum and enhance it by following some of the recommendations made by the students.

## LIMITATIONS

Our study did suffer from several limitations. The sample sizes were relatively small, and we were unable to include all of the students in the clerkship into the study because of logistical issues related to differing rotation start dates for students from various medical schools. Additionally, approximately two-thirds of the students had received previous ultrasound training.^15^ Thus, it is possible that our cohort of students scored better on the test than residents as a result of their previous ultrasound training, not because of the effectiveness of the ultrasound curriculum. Also, the qualitative data we received from the feedback survey had a moderate response rate. The potential exclusion of students who did not have a favorable opinion of the program could have skewed our results. Lastly, the residents who took the test to serve as the comparison group also suffered from a low response rate, as less than 50% of the residents submitted a completed version of the test to the investigators.

It is also worth noting that because of the limitations of designing research within the constraints of an established clerkship, we did not have a true control group of medical students who were tested but not exposed to the educational intervention. Lastly, while our data demonstrate that medical students were able to perform at a high level on a knowledge-based examination, the higher scores, while statistically significant, amounts to on average just one question on a 10-question exam. A clinical significance is likely negligible.

## CONCLUSION

Our study demonstrates that with a dedicated, integrated curriculum in POC ultrasound fourth-year medical students on an EM clerkship are able to perform similarly to EM residents on a knowledge-based exam. Further, our research shows that medical students find POCUS training to be a useful adjunct to the EM clerkship curriculum and that they feel the skills they acquire during this time will serve to benefit them on future clerkships and during residency training. Educators who are attempting to develop POCUS curricula for their existing EM clerkships would be wise to heed the advice of the students and include more than one scanning shift and also add POC echocardiography to the curriculum.

## Figures and Tables

**Table 1:** Student survey responses regarding the point-of-care ultrasound training program.

Question	Response
What specialty are you applying to?	EM (n=14), Internal Medicine (n=5), Family Medicine (n=3), Other (n=7): Surgery, Radiology, Neurology, Anesthesiology and Ophthalmology. See Figure 1.
Are you utilizing US on current rotations?	Yes (82.8%)
Are you utilizing US on Non-EM rotations?	Yes (58.6%)
Do you see yourself utilizing US in residency?	Yes (93.1%)
What did you find most useful about curriculum?	One-on-one instruction (55.2%)
What other applications would you have liked to learn about?	Echocardiography (24.1%); MSK (10.3%); Abscess (6.9%); DVT (6.9%)
How can we improve the curriculum?	Additional scanning shifts (51.7%); More evaluation of patients with pathology (24.1%)

*EM,* emergency medicine; *US,* ultrasound; *MSK,* musculoskeletal; *DVT,* deep vein thrombosis

**Table 2:** Multiple-choice ultrasound knowledge test scores.

Group	Mean* (SD)	Median	Minimum	Maximum
Medical students (n=45)	8.3 (1.2)	8.0	6.0	10.0
EM residents (n=20)	7.6 (1.1)	8.0	5.0	9.0

*SD,* standard deviation; *EM,* emergency medicine

* Mean scores were significantly different between students and residents t(63)=2.3, p=0.026.
